# Associations of the BDNF Val66Met Polymorphism With Body Composition, Cardiometabolic Risk Factors, and Energy Intake in Youth With Obesity: Findings From the HEARTY Study

**DOI:** 10.3389/fnins.2021.715330

**Published:** 2021-11-12

**Authors:** Gary S. Goldfield, Jeremy Walsh, Ronald J. Sigal, Glen P. Kenny, Stasia Hadjiyannakis, Michael De Lisio, Mathew Ngu, Denis Prud’homme, Angela S. Alberga, Steve Doucette, Diana B. Goldfield, Jameason D. Cameron

**Affiliations:** ^1^Healthy Active Living and Obesity Research Group, Children’s Hospital of Eastern Ontario Research Institute, Ottawa, ON, Canada; ^2^Department of Pediatrics, University of Ottawa, Ottawa, ON, Canada; ^3^School of Human Kinetics, University of Ottawa, Ottawa, ON Canada; ^4^Department of Kinesiology, McMaster University, Hamilton, ON, Canada; ^5^Department of Medicine, Cardiac Sciences and Community Health Sciences, Cumming School of Medicine, University of Calgary, Calgary, AB, Canada; ^6^Clinical Epidemiology Program, Ottawa Hospital Research Institute, Ottawa, ON, Canada; ^7^Centre for Healthy Active Living, Children’s Hospital of Eastern Ontario, Ottawa, ON, Canada; ^8^President and Vice Chancellor, University of Moncton, Moncton, NB, Canada; ^9^Department of Kinesiology, Concordia University, Montreal, QC, Canada; ^10^Department of Community Health and Epidemiology, Dalhousie University, Halifax, NS, Canada; ^11^Department of Life Sciences, Queen’s University, Kingston, ON, Canada; ^12^Department of Pharmacy, Children’s Hospital of Eastern Ontario, Ottawa, ON, Canada

**Keywords:** obesity, BDNF, gene, adolescents, Val66Met, energy intake, cardiometabolic risk

## Abstract

The brain-derived neurotrophic factor (BDNF) Val66Met polymorphism is functionally related to BDNF, and is associated with obesity and metabolic complications in adults, but limited research exists among adolescents. This study comparatively examined carriers and non-carriers of the BDNF Val66Met polymorphism on body composition, energy intake, and cardiometabolic profile among adolescents with obesity. The sample consisted of 187 adolescents with obesity; 99 were carriers of the homozygous Val (G/G) alleles and 88 were carriers of the Val/Met (G/A) or Met (A/A) alleles. Cardiometabolic profile and DNA were quantified from fasted blood samples. Body composition was assessed by magnetic resonance imaging (MRI). Compared to carriers of the homozygous Val (G/G) allele, carriers of the Val/Met (G/A) or Met/Met (A/A) variants exhibited significantly higher protein (*p* = 0.01) and fat (*p* = 0.05) intake, C-Reactive protein (*p* = 0.05), and a trend toward higher overall energy intake (*p* = 0.07), fat-free mass (*p* = 0.07), and lower HDL-C (*p* = 0.07) Results showed for the first time that among youth with obesity, carriers of the Val66Met BDNF Met-alleles exhibited significantly higher C-reactive protein and energy intake in the form of fat and protein compared to Val-allele carriers, thereby providing support for the possible role of BDNF in appetite, weight, and metabolic regulation during adolescence.

**Clinical Trial Registration:** http://clinicaltrials.gov/, identifier NCT00195858.

## Introduction

The high prevalence of pediatric obesity globally (Di Cesare et al., 2019) poses a serious public health concern given the high and growing social, economic, and health burden associated with this chronic disease (Wellman and Friedberg, 2002), and that obesity and associated health risks track from childhood into adulthood. Independent of geographical location, weight gain leading to obesity is the result of a complex combination of genetic and environmental factors that ultimately result in energy intake exceeding energy expenditure (Farooqi and O’Rahilly, 2007). Although it is estimated that around 5% of severe obesity is dependent on the influence of a single gene (e.g., MC4R), there are heritability estimates between 40 and 70% depending on the populations studied (Farooqi and O’Rahilly, 2007). Consequently, obesity is a highly heritable and heterogeneous disorder. Body mass index (BMI) is used as a criterion for measuring overweight (BMI 25–29.2 kg/m^2^) and obesity (BMI ≥ 30 kg/m^2^) in adults, and growth curves based on the same criteria that correspond to ≥ 85th BMI percentile (overweight) or ≥ 95th BMI percentile (obesity) for age and sex have typically been used for children and youth. Several candidate genes have been identified as being positively associated with BMI and obesity, including the fat mass and obesity associated gene (FTO) (Zhao et al., 2014), and more recently, the brain derived neurotrophic factor (BDNF) gene (Zhao et al., 2009).

Brain-derived neurotrophic factor is a protein that exerts pleiotropic effects on the brain and peripheral tissue (Marosi and Mattson, 2014), and is synthesized from a precursor protein (pro-BDNF) (Carlino et al., 2011). BDNF is one of the most expressed neurotrophins in the brain, with primary actions in the hippocampus, cerebral cortex, cerebellum, hypothalamus, and brainstem (Egan et al., 2003). Although primarily recognized for its role in facilitating the proliferation, differentiation, and overall survival of neurons in the CNS (Egan et al., 2003), the actions of BDNF have garnered interest in the study of the central control of food intake due to abundant expression in brain areas involved in appetite regulation (Lebrun et al., 2006). BDNF is also involved in regulating metabolic functions, such as fat oxidation and glucose utilization (Tsuchida et al., 2001; Yamanaka et al., 2007; Matthews et al., 2009), and has been shown to be downregulated in those with obesity and type 2 diabetes (Krabbe et al., 2007; Zhao et al., 2009). Animal models have shown that the deletion of BDNF in the mouse brain results in obesity, hyperphagia, and impaired locomotor activity (Kernie et al., 2000), and in humans the loss of one copy of the BDNF gene is also associated with hyperphagia, severe obesity, and impaired cognitive function (Gray et al., 2006).

A common single nucleotide polymorphism in the human BDNF gene (rs6265) causing an amino acid substitution of Valine to Methionine at residue 66 (i.e., Val66Met) has recently garnered heightened interest as a functional polymorphism of the BDNF gene. Specifically, the Met variant is thought to be responsible for abnormal intracellular packaging of the precursor of BDNF (pro-BDNF) as well as decreased production/expression of mature BDNF (Egan et al., 2003). Thus, the Met allele has been identified as a hypofunctional BDNF allele, whereas the Val allele is associated with normal packaging and expression of BDNF. The so-called hypofunctioning Met allele was positively associated with BMI in a large sample of human adults (Shugart et al., 2009) and children (Skledar et al., 2012; Martínez-Ezquerro et al., 2017), but other studies reported no associations in adults (Friedel et al., 2005) and children (Arija et al., 2010; Vidovic et al., 2020). Moreover, other studies have found opposite results, whereby the Met allele was associated with lower BMI in healthy adults (Sustar et al., 2016), adolescents (Kalenda et al., 2018), and lower fasting (Vidovic et al., 2020) and post-prandial glucose levels (Kalenda et al., 2018). Similarly, there is some evidence, albeit limited, to suggest that the minor Met allele is associated with increased caloric intake in children (Kalenda et al., 2018), but stronger evidence to suggest that the Met allele is associated with disordered eating, such as binge-eating (Gratacòs et al., 2007), consistent with animal research showing BDNF suppression is associated with hyperphagia (Kernie et al., 2000). Interestingly, despite the detrimental effects of the Met allele, the Val66Met polymorphism is highly conserved in humans and may offer selective advantages. Indeed, the Val66 (Val/Val) BDNF prodomain ligand facilitates long-term depression in the hippocampus (Zanin et al., 2017), which may have implications for neurocognitive (Voineskos et al., 2011), appetitive and metabolic disorders (Zanin et al., 2017).

The purpose of this study was to examine whether carriers of the BDNF Val66Met polymorphism Met-allele (Val/Met -G/A alleles or Met/Met-A/A alleles) differed from carriers of the homozygous Val/Val (G/G) alleles on anthropometrics, cardiometabolic risk factors, and energy intake in a sample of adolescents living with overweight and obesity. It was hypothesized that BDNF Met-allele carriers would exhibit greater adiposity, higher energy intake, and poorer cardiometabolic health markers when compared to the homozygous Val-allele carriers.

## Materials and Methods

### Participants

The current study uses a cross-sectional design representing a secondary analysis of baseline data from the Healthy Eating Aerobic and Resistance Training in Youth (HEARTY) trial, which examined effects of exercise training on percent body fat as the primary outcome (Sigal et al., 2014), and other physical and mental health outcomes in youth with overweight and obesity (Alberga et al., 2015; Goldfield et al., 2015; McNeil et al., 2017), including quality of life (Goldfield et al., 2017). The sample that had given informed consent for genetic analysis and had data of interest consisted of 187 participants out of the full sample of 304 (62% of the full baseline HEARTY sample), but sample characteristics between this sub-sample and the full HEARTY sample did not differ significantly (data not shown).

Inclusion criteria for participants in the HEARTY study included: Being post-pubertal (Tanner stages IV-V), aged 14–18 years, and with BMI > 95th percentile for age/sex and/or ≥ 85th percentile for age/sex with at least one additional diabetes or cardiovascular disease (CVD) risk factor, as described elsewhere (Alberga et al., 2012). Exclusion criteria included participation in regular or structured exercise or sport activities more than twice a week for more than 20 min during the previous 4 months, diabetes mellitus, use of any performance enhancing medication, significant weight change (increase or decrease of ≥ 5% body weight) during the 2 months before enrollment, pregnancy at the start of the study, activity restrictions due to disease (unstable cardiac or pulmonary disease or significant arthritis), and other illness (e.g., eating disorders/clinical depression) judged by the participant or study physician to make participation in this study inadvisable.

Most of the sample (74%) were of European descent. Eighty-three percent reported coming from parents who completed some university or community college. All participants provided informed consent and/or assent. A parent or guardian was asked to co-sign the assent form for any participants below the age of 16 years, while participants aged 16 years or older provided their own informed consent. This study received approval from the Research Ethics Boards at the Children’s Hospital of Eastern Ontario (protocol #05/04E) and the Ottawa Hospital Research Institute (protocol #2004219-01H). Study protocols conformed to the Declaration of Helsinki (World Medical Association, 2013).

### Design and Procedure

For this cross-sectional study conducted at the baseline assessment of the HEARTY study, the research coordinator performed a complete medical, drug, and physical activity history as well as a physical examination. Clinical interviews were also performed to assess inclusion/exclusion criteria, as well as medical and developmental history as described elsewhere (Alberga et al., 2012). Sociodemographic characteristics, pubertal status, lifestyle behaviors, and free-living energy intake were completed by self-reported measures in the laboratory, while body composition was quantified using objective measures in the laboratory and MRI (Alberga et al., 2012). The study was conducted from March 2005 to June 2011.

### Measurements

#### Primary Independent Variable

BDNF Val66Met Polymorphism: Val (G/G), Met (A/A), and Val/Met (G/A) alleles. DNA was extracted from 12-hour (overnight-fasting) blood samples of approximately 20 mL of venous blood taken in the morning from a forearm or antecubital vein, and stored in a freezer at −80°C. Isolation of genomic DNA from buffy coat samples was completed following manufacturer’s instructions (FlexiGene DNA Kit (250), Qiagen, Cat No. ID: 51206, Germany). DNA concentration was measured using a spectrophotometer (NanoDrop^TM^ 2000, Thermo Scientific, Waltham, MA, United States). PCR reactions were carried using the following primers (P1: CCTACAGTTCCACCAGGTGAGAAGAGTG, P2: TCATGG ACATGTTTGCAGCATCTAGGTA, P3: CTGGTCCTCATCCA ACAGCTCTTCTATAAC and P4: ATCATTGGCTGACACTTT CGAACCCA), and BDNF genotyping followed the procedures described by Sheikh et al. (2010). The four primers amplify two allele-specific amplicons (253 and 201 bp) and the entire region as an internal control. The PCR reaction was carried out in a 25 μl reaction volume including: 25 ng of genomic DNA template, the primers, 100 mmol/l of dNTP (Invitrogen, CA, United States), 3 mmol/l of MgSO4 (Invitrogen, CA, United States), 1X PCRx Amplification Buffer (Invitrogen, CA, United States), 1X PCRx Enhancer Solution (Invitrogen, CA, United States) and 1 U Taq DNA Polymerase (Invitrogen, CA, United States). The PCR cycling conditions used an initial denaturation temperature of 94°C for 5 min, followed by 30 cycles of 94°C for 45 s, 62.5°C for 60 s and 72°C for 60 s and a final extension step of 5 min at 72°C. PCR amplicons were resolved on a 1.5% polyacrylamide gel, stained with BlueJuice^TM^ Gel Loading Buffer (Invitrogen, CA, United States) and visualized on the ChemiDoc^TM^ Gel Imaging System (Bio-Rad Laboratories, Mississauga, ON, Canada).

### Dependent Variables

#### Body Composition

Height and weight were measured with a stadiometer and balance-beam scale, respectively, with participants wearing light clothing and no shoes. BMI was quantified by weight in kilograms divided by height in meters-squared. Waist circumference was measured at a level midway between the lowest rib and the top of the iliac crest, as previously described (Alberga et al., 2012). Body composition was assessed by MRI with a 1.5-T system (EchoSpeed, signal 11 version; GE Medical Systems). Participants lay prone for whole-body cross sectional images using protocols by Ross et al. (1992). The MRIs were analyzed using Slice-OMatic^TM^ software, version 4.3; (Tomovision, Magog, QC, Canada). Fat-free mass (FFM) is defined as total lean tissue mass, including all fat-free skeletal muscle, organs, intestines, and bones, without adipose tissue, while fat mass (FM) represents the amount of visceral and subcutaneous adipose tissue. Percent body fat was calculated by dividing the amount of FM by total body mass (i.e., FM + FFM) × 100.

#### Cardiometabolic Risk Factors

Twelve-hour (overnight-fasting) blood samples of approximately 20 mL of venous blood were taken in the morning from a forearm or antecubital vein and were stored in a freezer at −80°C. The lipid profile measurements included triglycerides (mmol/L), total cholesterol (mmol/L), and High Density Lipoprotein (HDL-C, mmol/L), which were measured by using enzymatic methods on a Beckman-Coulter LX20 analyzer (Beckman instruments, Brea, CA, United States), while LDL-C concentrations were calculated by using the Friedewald equation (Friedewald et al., 1972). Total cholesterol/HDL-C ratio was derived from measured values. High-sensitivity C-Reactive Protein (C-RP) was measured using highly sensitive Near Infrared Particle Immunoassay rate methodology (Beckman Coulter Unicel DxC600 Synchron Clinical System, and Beckman reagents Beckman Coulter Inc.). Blood pressure (BP) was measured manually using a mercury sphygmomanometer on the left arm after 4 min of rest, with subjects sitting with their back supported. Three BP measurements were taken at 1-min intervals; the mean of the final two measures of BP was used for the analysis.

#### Energy Intake: 3-Day Food Logs

Under the supervision of a registered dietitian, energy intake (EI) was assessed using 3-day self-reported food logs. Regarding the reliability of this measure, in a recent paper examining the magnitude of EI misreporting, it was determined that there was no significant difference between the medians of percentage of misreporters when comparing three of the main methods of self-reported food intake: 24 h recall, 3- and 7-day food logs, and weighed food records (underestimation of EI was 13.4, 12.2, and 18.0%, respectively; Poslusna et al., 2009). Adolescents were asked to complete their 3-day food logs in real time, right after eating, over two weekdays and one weekend day prior to the visit with the dietitian. Participants were instructed to record the quantity or weight of all food and beverages consumed and to record methods of food preparation, brand names and ingredients of foods, and recipes of mixed dishes when possible. Completed food logs were discussed between the participant and the dietitian during the in-person visit for clarification of food details and amounts. Participants were counseled on measuring portion sizes using food models supplied with a handout that described in detail how to measure food portions. If participants did not have access to measuring tools (cups, spoons, etc.), they were advised to follow the Canadian Diabetes Association’s Handy Portion Guide included in the handout^1^. The food logs were analyzed with food composition analysis software (The Food Processor SQL 2006, ESHA Research, Salem, OR, United States) to determine total energy intake (average daily kcal) and separate macronutrient intake (grams) for carbohydrate, protein and fat for each participant. In most cases the days recorded were consecutive, including one weekend day and two weekdays, although some of the weekday reporting included non-consecutive days. Intake of each nutrient class was averaged across the 3 days for analysis.

### Descriptive Variables

#### Demographic and Developmental Variables

Background socio demographic information was obtained from all participants, including age, sex, ethnicity, and highest level of parental education as measured by self-report. The Tanner pubertal staging system was used to assess pubertal stage, although all participants were post-pubertal.

#### Physical Activity

Self-reported physical activity duration was calculated based on the question, “On average, how long do you participate in some sort of physical activity (PA) each day?” with physical activity being cumulative, not consecutive. Six response options were provided: 1 ≤ 5 min, 2 = 5–15 min, 3 = 15–30 min, 4 = 30–45 min, 5 = 45–60 min, and 6 ≥ 60 min. Physical activity duration (minutes/day) was used as a covariate in the analysis.

#### Traditional Screen Time Behavior

Traditional screen time behavior was assessed by questionnaire evaluating how much time, in hours per day, participants spent watching television, playing seated/inactive video games (excluding computer games), and using the computer for recreational reasons (excluding school work and including computer games). The questions on screen time assessed usage on both weekday and weekend days. The three types of screen behaviors (i.e., TV, seated video games, and recreational computer use) were aggregated and then averaged over weekend and weekdays to make up a measure of total screen time duration/day (i.e., weekday screen time/5 + weekend screen time/2 = average total screen-time/day). Similar methods of self-reported screen time have been shown to have acceptable reliability and validity in adolescents (Leatherdale and Harvey, 2015).

### Statistical Analyses

All outcome variables were examined for normality. All body composition and cardiometabolic variables were positively skewed while energy intake variables were normally distributed, except for sugar-sweetened beverage intake. All distributions were successfully normalized with either logarithmic or square root transformations based on criteria previously described (Tabachnick and Fidell, 2007). Baseline characteristics of the sample were computed and are presented in Table 1 using means and standard deviations for continuous data and frequencies and percentages for categorical data. Since the frequency of the homozygous Val66Met Met/Met (A/A) genotype is low (1–8%) in populations primarily comprised of European descent (Shen et al., 2018), we combined this group (*n* = 6) with the carriers of the Val/Met (G/A) alleles. These carriers of the Met-alleles (*n* = 88) were compared to carriers of the homozygous Val/Val (G/G, *n* = 99) genotype on variables of interest using independent *t*-tests for continuous data or Chi-Square tests for categorical data. Because there were no group differences on demographic, anthropometric, or behavioral variables, group differences on outcomes of interest (body composition, energy intake, and cardiometabolic profile) were evaluated by univariate statistics (independent *t*-tests) on transformed variables. This was done to conserve statistical power rather than statistically controlling for these variables using multivariate modeling, which would reduce power unnecessarily. It is important to note that untransformed data were reported in Tables to aid the interpretation of behavioral, physiological, and clinical outcomes, but *p*-values reported were based on analyses with transformed data. Setting power at 0.80 and assuming a two-tailed alpha of 0.05, we could detect a significant group difference reflecting a small to medium effect size (Cohen’s *d* = 0.40) in outcomes of interest using independent *t*-tests with a sample of 97 participants per group; thus, our sample obtained provided adequate, albeit not strong, power to assess our objectives (Cohen, 1977). The genotype distribution of the current sample was within Hardy–Weinberg equilibrium based on a Chi-Square test (*p* = 0.65) in comparison to a population of youth with obesity of European descent (Skledar et al., 2012). Statistical significance was defined as a two-tailed alpha < 0.05. Analyses were conducted using SPSS, version 24.

**TABLE 1:** Characteristics of the sample by BDNF Val66Met genotype.

	Val66Met Genotype		
Variable	Homozygous Val/Val (G/G Carriers) (*n* = 99) Mean (SD)	Heterozygous Val/Met (G/A) (*n* = 82) or Met/Met (A/A) (*n* = 6) Carriers Mean (SD)	*P*-value
Age	15.5 (1.5)	15.8 (1.3)	0.16
Screen Time (hours/day)	5.5 (3.0)	6.0 (2.9)	0.24
Percent Body Fat (MRI)	50 (5.8)	49 (5.9)	0.25

	***n* (%)**	***n* (%)**	

**Gender**			
Male	29 (29.3%)	33 (37.5%)	0.28
Female	70 (70.7%)	55 (62.5%)	
**Ethnicity**			
White	71 (71.7%)	68 (77.3%)	0.39
Non-White	28 (28.3%)	20 (22.7%)	
**Parent Education (Highest)**			
High School	19 (19.2%)	13 (14.8%)	0.44
College/University	80 (80.8%)	75 (85.2%)	
**Tanner Stage**			
4	33 (33.3%)	20 (37.7%)	0.14
5	66 (66.7%)	68 (77.3%)	
**Physical Activity (minutes/day)**			
<15 min	31 (31.6%)	31 (35.2%)	0.94
15–30 min	26 (26.5%)	23 (26.1%)	
30–60 min	24 (24.5%)	15 (17.1%)	
>60 min	17 (17.4%)	19 (21.6%)	

## Results

Table 1 shows the Val66Met polymorphism allele frequency breakdown for the sample. Only six participants were carriers of the homozygous (A/A) Met allele, with approximately equal proportions of participants carrying either the homozygous Val (G/G) or Val/Met (G/A) variants. There were no significant group differences on age, sex, parental education, ethnicity, pubertal stage, adiposity, physical activity, or screen-time. The sample was, on average, 15.5 years old, living with obesity, primarily White and coming from well-educated parents/families. Approximately 67% of the sample was female. On average, the sample of adolescents was not physically active, and engaged in 5.6 h of total screen time per day.

Table 2 shows that compared to carriers of the Val66Met homozygous Val (G/G) alleles, carriers of the Met (G/A or A/A) alleles reported significantly higher C-Reactive protein [*t*(1,185) = 1.96, *p* = 0.05], energy intake in the form of protein [*t*(1,184) = 2.46, *p* = 0.01] and fat, [*t*(1,184) = 1.96, *p* = 0.05], and a trend toward higher overall energy intake [*t*(1,184) = 1.81, *p* = 0.07], fat-free-mass [*t*(1,181) = 1.82, *p* = 0.07], and lower HDL-C [*t*(1,183) = 1.82, *p* = 0.07]. No other genotypic differences were found on indicators of body composition, cardiometabolic risk factors or energy intake.

**TABLE 2:** Body composition, cardiometabolic risk, and energy intake by BDNF gene carriers.

	Val66Met Genotype		
	Homozygous Val/Val (G/G Carriers) (*n* = 99) Mean (SD)	Heterozygous Val/Met (G/A, *n* = 82) or Met/Met (A/A, *n* = 6) Carriers	*P*-value
**Body Composition:**			
Percent Body Fat	50.0 (5.8)	49.0 (5.9)	0.29
Fat-free mass (kg)^a^	47.6 (8.4)	50 (8.9)	0.07
Waist-to-hip ratio (cm)^b^	0.82 (0.07)	0.82 (0.07)	0.94
Weight (kg)^a^	98.7 (16.7)	101.1 (18.6)	0.40
BMI (kg/m^2^)^c^	34.9 (4.6)	35.2 (4.6)	0.57
**Cardiometabolic:**			
HDL-C (mmol/L)^d,e^	1.12 (0.25)	1.06 (0.29)	0.07
LDL-C (mmol/L)^e,f^	2.62 (0.76)	2.56 (0.65)	0.56
C-Reactive Protein (mg/L)^g^	3.1 (3.2)	4.6 (5.7)	**0.05**
Triglycerides (mmol/L)^e^	1.3 (0.6)	1.4 (0.6)	0.34
Systolic BP (mm/Hg)^h^	113.2 (9)	114.7 (10.2)	0.33
Diastolic BP (mm/Hg)^h^	75.1 (6.9)	74.9 (7.2)	0.74
**Energy Intake**			
Total Intake (kcals/day)^i^	2,156 (575.8)	2,331 (741.2)	0.07
Total Fat Intake (g)^j^	81.9 (29.1)	91.2 (38.3)	**0.05**
Total Protein Intake (g)^j^	81.1 (26.4)	90.9 (27.8)	**0.01**
Total Carb Intake (g)^j^	279.9 (77.5)	290.7 (100.9)	0.41
Sweetened drinks (kcals)^i^	231.2 (267.2)	217.1 (224.8)	0.78

*^a^kg, kilograms; ^b^cm, centimeters; ^c^BMI, Body Mass Index; ^d^HDL-C, high density lipoprotein-cholesterol; ^e^mmol/L, millimoles per liter; ^f^LDL-C, low density lipoprotein cholesterol; ^g^mg/L, milligrams per liter; ^h^mm/Hg, millimeters of mercury; ^i^kcals, kilocalories; ^j^g, grams.*

## Discussion

The current study comparatively examined if adolescents with obesity carrying different variants of the BDNF Val66Met polymorphism differed on body composition, energy intake and cardiometabolic profile. We found that carriers of one or two copies of the Met alleles (G/A or A/A variants) exhibited higher energy intake in the form of protein and fats, and higher C-Reactive protein compared to carriers of the homozygous Val alleles.

Although all participants in our study were living with overweight or obesity, our findings that the BDNF polymorphism could not differentiate carriers of Val vs. Met variants on BMI is consistent with studies that included youth with and without obesity from Spain (Arija et al., 2010), Mexico (León-Mimila et al., 2013) and Serbia (Vidovic et al., 2020). Similarly, Friedel et al. (2005) reported that no differences in BDNF allele frequency were observed in German children and youth with severe obesity, underweight students, and normal weight controls. However, other studies have found carriers of the hypofunctioning Met allele of the Val66Met polymorphism showed greater adiposity based on BMI percentiles or BMI-for-age z-scores in population-based cohorts in European American youth (Zhao et al., 2009), Chinese youth (Wu et al., 2010), Croatian youth (Skledar et al., 2012) and Mexican youth (Martínez-Ezquerro et al., 2017), yet Met-carriers were found to have lower standardized BMI in other population-based samples of European youth (Kalenda et al., 2018). Similarly, Sustar et al. (2016) found that healthy adult carriers of the Met/Met allele had lower BMI, but no association was found between the BDNF Val66Met genotypes and weight status in adults with coronary heart disease, indicating the role that the Met/Met allele plays in obesity may differ by population. Unlike previous studies that have solely relied on BMI, our study is the first to investigate the association between BDNF polymorphism and body composition using MRI, yielding a higher level of precision in quantifying fat mass and fat-free mass, noted to be a more reliable predictor of obesity-related complications than BMI-based measures (Mitra et al., 2017). Interestingly, we found that despite no differences in body weight, BMI, or fat-mass, carriers of the Met-alleles of the Val66Met polymorphism showed a trend toward greater fat-free mass compared to the homozygous Val (G/G) carriers, while another study in pediatrics showed lower central adiposity as measured by waist circumference among Met-allele carriers (Xi et al., 2013). Future research should confirm these initial findings to determine not only the associations between the BDNF genotype and the obesity phenotype as measured by BMI, but also if meaningful differences in rigorous measures of body composition exist between carriers of different Val66Met alleles, and how these differences may be related to metabolic and feeding-related outcomes.

Brain-derived neurotrophic factor has been shown to play an integral role in regulating appetite in both humans (Gray et al., 2006; Marosi and Mattson, 2014) and animals (Kernie et al., 2000; Lebrun et al., 2006), and exogenous administration of BDNF has hypophagic effects (Rios, 2013), while BDNF deletion has hyperphagic effects leading to severe obesity (Gray et al., 2006). Moreover, the BDNF gene is functionally associated with an estimated 33% reduction in available BDNF, and animal models show that the anorexigenic effect of BDNF is mediated through its modulating impact on the melanocortin/leptin system, as well as the dopamine and serotonin neurotransmitter systems (Bariohay et al., 2005). Consistent with these animal and human studies, we found youth with the hypofunctioning Met-alleles reported greater energy intake in the form of fat and protein, and although not statistically significant, we observed trends (*p* = 0.07) for higher overall caloric consumption compared to homozygous Val-allele carriers. The energy surplus of approximately 175 Kcal observed by Met-allele carriers, if maintained over time and with no compensatory increase in energy expenditure or metabolic adaptations, could lead to weight gain consistent with research in adult humans showing that genetically-driven decreases in BDNF are associated with disordered eating (Gratacòs et al., 2007) and hyperphagia (Gray et al., 2006). Nevertheless, it should be noted that Met-allele carriers also showed a trend toward higher fat-free mass (2.4 kg), which has been shown to be a strong predictor of higher energy needs and consumption (Cameron et al., 2016). However, whether the higher fat-free mass, which may be partly due to higher body weight (1.4 kg), would completely offset the higher energy intake cannot be ascertained in our study, highlighting the need for future studies to include rigorous measures of both energy expenditure and energy intake to better evaluate the risk of weight gain and obesity.

We also found the hypofunctioning BDNF Met-allele carriers of the Val66Met polymorphism not only reported higher energy intake but also exhibited approximately 50% higher fasted C-reactive protein (CRP) levels and a trend toward lower HDL-C levels in the blood compared to Val-carriers. This is clinically meaningful given CRP and HDL-C are among the most reliable biomarkers of chronic inflammation and cardiometabolic disease (van Holten et al., 2013). The higher CRP levels associated with the Met-allele carriers of the BDNF polymorphism provide evidence that the impact of BDNF on the pathophysiology of cardiovascular disease that has been recognized in adults (Lorgis et al., 2009; Pius-Sadowska and Machaliński, 2017) may actually occur during adolescence. In support of this relationship, human Val/Met carriers with unstable angina have significantly elevated plasma CRP compared to Met/Met carriers with unstable angina (Jiang et al., 2009). Relatedly, we found that Met-allele carriers exhibited lower HDL-C compared to Val/Val carriers, consistent with findings among adults from China (Peng et al., 2017), while another study in adults showed that homozygous Met-allele carriers were 2.8 times more at risk of metabolic syndrome, an association that remained after adjustment for BMI (Rana et al., 2019).

Very few other pediatric studies have investigated relations between BDNF genotype, energy intake, and cardiovascular disease risk factors. Our findings are somewhat consistent with Kalenda et al. (2018), who found that, in post-pubertal (but not pre-pubertal) youth, Met-allele carriers also reported increased caloric, carbohydrate, and protein intake. Interestingly, this pattern of ingestive behavior in the Kalenda et al. (2018) study was associated with lower post-prandial glucose levels and HbA1C among Met-allele carriers. Also, a recent study in 308 Serbian adolescents with a mean BMI in the healthy range showed that Met-allele carriers exhibited lower fasted blood glucose levels compared to Val-carriers, an effect that was driven by females with no association in males (Vidovic et al., 2020). These findings provide evidence in childhood that BDNF appears to impact energy intake, macronutrient preference, and glucose regulation, previously established primarily in animal research (Kernie et al., 2000; Lebrun et al., 2006). It should be noted that differences in the pattern of results between our HEARTY study and those of Kalenda et al. (2018) and Vidovic et al. (2020) may be due, in part, to differences in sample characteristics. For example, our study was comprised of inactive, post-pubertal Canadian adolescents who were predominantly female (74%), White (67%) and coming from university-educated parents (83%). Other study samples were comprised of more socio economically diverse and more ethnically homogeneous (i.e., White) European children and adolescents recruited from the community who were pre- and post-pubertal, and who included youth with and without obesity, or who were at risk of eating disorders (Arija et al., 2010; Skledar et al., 2012; Kalenda et al., 2018; Vidovic et al., 2020). However, it should be noted that sociodemographic characteristics such as age, sex, parent education, ethnicity, or pubertal status did not differ by BDNF genotype in our sample, and therefore is unlikely to explain observed differences. Differing results between studies could also be due to population genetic differences in the Val66Met polymorphism (Shen et al., 2018). Nevertheless, our results showing that BDNF genotypes associated with suppression of mature BDNF levels *via* Trk are also associated with increased CRP levels is consistent with our previous work showing that exercise-induced increases in serum BDNF levels are associated with reduced cardio metabolic risk in youth with obesity (Walsh et al., 2018). These findings are also consistent with adult studies and animal research implicating BDNF in the regulation of systemic inflammation and glucose utilization and control (Kernie et al., 2000; Jiang et al., 2009; Stein et al., 2017).

Several molecular mechanisms are offered to explain our metabolic findings. Murine models of the human Val66Met BDNF polymorphism suggest that this pro-inflammatory phenotype is mediated, in part, by hypercoagulability of platelets and alterations in platelet-BDNF release dynamics (Stein et al., 2017). Indeed, BDNF activates the TrkB receptor leading to vascular endothelial cell integrity, survival, and function (Donovan et al., 1995; Prigent-Tessier et al., 2013), and the formation of cardiac vasculature (Emanueli et al., 2014). BDNF functions as an angiogenic regulator, promoting angiogenesis (Kermani et al., 2005). Alterations in platelet-BDNF interactions with the vascular endothelium coupled with a pro-inflammatory state may contribute to the increased risk of CVD associated with the hypofunctioning BDNF polymorphism *via* TrkB. However, it should be noted that BDNF is initially synthesized in the endoplasmic reticulum as its precursor protein, preproBDNF, which becomes proBDNF, which is then converted by extracellular proteases to mature BDNF (Ethel and Ethel, 2007; Yoshida et al., 2013; László et al., 2019) ProBDNF is biologically active: it mediates its actions through binding to low-affinity p75Ntr, having an antagonistic effect compared to matured BDNF. This reduces spine complexity and density (Zagrebelsky et al., 2005) and promotes neuronal cell death (Teng et al., 2005). Of particular importance are the BDNF gene precursors, such as the Val66 prodomain BDNF and Met66 prodomain BDNF (Zanin et al., 2017), which are secreted ligands by neurons that can exert potential functions when the BDNF gene is active. The Met-variant prodomain BDNF acutely alters neuronal morphology, inducing changes in neuron growth cones (Anastasia et al., 2013). This alteration in processing of Met prodomain BDNF may represent another possible mechanism that explains our findings that Met-allele carriers exhibited greater energy intake and metabolic risk compared to Val-allele carriers. It is noteworthy, however, that the Val66Met polymorphism is highly conserved and the Val66 prodomain BDNF ligand has been shown to reduce dendritic spine density in hippocampal neurons and facilitates long-term hippocampal depression (Guo et al., 2016), an effect that is blunted by the Met prodomain BDNF (Mizui et al., 2016). This highlights that the Met66 prodomain ligand may confer selective advantages to hippocampal structure and plasticity, which may also explain, in part, findings that the Met-allele carriers have exhibited lower cardiometabolic disease risk in children (Kalenda et al., 2018; Vidovic et al., 2020), lower BMI in healthy adults (Sustar et al., 2016), and fewer CVD events and milder CVD severity in adult cardiac patients (Jiang et al., 2017).

Our study has several strengths and limitations that are important to acknowledge. Firstly, our sample was comprised of inactive, post-pubertal youth living with overweight or obesity presenting for weight loss, who were primarily White, female, and coming from well-educated parents, so results may not be generalizable to all adolescents with or without obesity. Secondly, our data are cross-sectional in nature, thus causality or directionality cannot be inferred, highlighting the need for future studies to incorporate longitudinal designs with multiple follow-ups to better establish directionality. Thirdly, we assessed energy intake based on standardized self-reported food logs, and despite being verified with a registered dietitian, reporting bias cannot be discounted. Although there were no differences between carriers and non-carriers of the Val66Met-allele on demographic, anthropometric or lifestyle factors, it is possible additional environmental factors (e.g., stress, trauma, depression etc.) that were not accounted for in our design and analysis may have impacted the results *via* gene-environment interactions given their relationship with BDNF (Zhao et al., 2018). Additionally, although our sample size provided adequate power to detect medium-sized effects, it is considered somewhat small for genetic studies and this may have limited our statistical power to detect smaller effects, as well as examine sensitivity analyses such as the moderating effects of gender or ethnicity. Due to the novelty of our study and paucity of research in pediatrics, we did not want to be overly conservative by controlling for multiple comparisons which would have increased the odds of not detecting group differences when they exist (i.e., Type II error). However, future research aimed at replicating these findings may wish to adjust for multiple comparison to protect against the possibility of increased odds of obtaining significant findings by chance (Type I error). Finally, we did not measure serum BDNF, appetite, or energy expenditure, which should be included in future research to provide a better understanding of how the BDNF polymorphism functionally impacts serum BDNF levels and how this is associated with energy balance, body composition, and metabolic comorbidities in youth. Strengths of our study included its novelty, and a high risk clinical sample of youth with obesity who exhibit greater incidence of BDNF-related complications such as neurocognitive deficits (Liang et al., 2016) and metabolic dysregulation (Krabbe et al., 2007) compared to peers without obesity. Additionally, unlike other studies, we did not solely rely on BMI-based measures of body composition, but instead used MRI, which provides more precise measurement and more reliable associations with obesity-related complications than BMI (Mitra et al., 2017), strengthening the internal validity of the findings.

In conclusion, we found that carriers of the Met-alleles of the BDNF Val66Met polymorphism exhibited significantly and clinically meaningfully (50%) higher CRP and energy intake in the form of fats and protein, with trends for higher overall energy intake, fat-free mass, and lower HDL-C compared to carriers of the homozygous Val-allele. Given youth with obesity are at increased risk of cardiometabolic disorders in adulthood, and BDNF has been implicated in energy intake and metabolic dysregulation as described above, future research is needed to verify our findings and determine whether carriers of this BDNF polymorphism are genetically predisposed for increased energy intake, weight gain and metabolic complications. In the era of personalized medicine, this information is essential to inform early intervention strategies designed to optimize pediatric obesity and chronic disease prevention and management.

## Data Availability Statement

The raw data supporting the conclusions of this article will be made available by the authors, without undue reservation.

## Ethics Statement

The studies involving human participants were reviewed and approved by Research Ethics Board at the Children’s Hospital of Eastern Ontario (protocol #05/04E) and the Research Ethics Board at the Ottawa Hospital Research Institute (protocol #2004219-01H). Written informed consent to participate in this study was provided by the participants’ legal guardian/next of kin.

## Author Contributions

GG conceived of the study and participated in its design and coordination, and drafted the manuscript. JW, SH, and DG helped to draft and critically review the manuscript. RS, GK, and DP conceived of the study and participated in its design and coordination, and helped to draft and critically review the manuscript. ML oversaw the genotyping and helped to draft and critically review the manuscript. MN conducted the genotyping, helped to draft and critically review the manuscript. AA helped with data collection and to draft and critically review the manuscript. SD performed the statistical analyses, and helped to draft and critically review the manuscript. JC helped conceive of the study and its design and coordination, and helped to draft and critically review the manuscript. All authors have read and approved the final version of the manuscript, and agree with the order of presentation of the authors.

## Conflict of Interest

The authors declare that the research was conducted in the absence of any commercial or financial relationships that could be construed as a potential conflict of interest.

## Publisher’s Note

All claims expressed in this article are solely those of the authors and do not necessarily represent those of their affiliated organizations, or those of the publisher, the editors and the reviewers. Any product that may be evaluated in this article, or claim that may be made by its manufacturer, is not guaranteed or endorsed by the publisher.

## Footnotes

1
https://www.diabetes.ca/DiabetesCanadaWebsite/media/Managing-My-Diabetes/Tools%20and%20Resources/handy-portion-guide.pdf?ext=.pdf
